# The Adverse Associations of Classrooms’ Indoor Air Quality and Thermal Comfort Conditions on Students’ Illness Related Absenteeism between Heating and Non-Heating Seasons—A Pilot Study

**DOI:** 10.3390/ijerph18041500

**Published:** 2021-02-05

**Authors:** Shihan Deng, Bin Zou, Josephine Lau

**Affiliations:** 1 Faculty of Civil Engineering and Mechanics, Kunming University of Science and Technology, Kunming 650050, China; sdeng@kust.edu.cn (S.D.); luffy8187@163.com (B.Z.); 2 Scott-Campus/Durham School of Architectural Engineering and Construction/College of Engineering, University of Nebraska-Lincoln, Lincoln, NE 68128, USA

**Keywords:** absence rate, attendance, seasonal variation, the Midwest U.S.

## Abstract

(1) The association of the indoor environmental conditions in classrooms with illness-related absenteeism (IRA) was not well investigated. In addition, studying the association between heating and non-heating seasons were very limited; (2) To fill this knowledge gap, a research team collected various indoor air quality (IAQ) and thermal comfort conditions (TC) of 85 elementary classrooms in two school districts from the Midwestern United States throughout an academic year; in total, 255 classroom visits were performed. A negative binomial regression model was implied to associate the classroom’s IAQ and TC with IRA, separating for heating and non-heating seasons; (3) During non-heating season, a 3% increase of IRA was estimated with 1,000,000-counts/L increase of particles that had a diameter less than 2.5 μm (PN2.5); during the heating season, a 3% increase of IRA were expected with 100 ppm increase of room averaged CO_2_ concentration; and (4) These results suggested that the IAQ and TC factors could associated with IRA differently between heating and non-heating seasons.

## 1. Introduction

Schools are the places where children spend most of their time during their childhood other than their homes. Children are more vulnerable to adverse indoor environmental quality (IEQ) due to having a more rapid breathing rate when compared to adults, and also to their still-growing body and still-developing organs. Teachers, parents, school officials, and researchers are becoming more aware of the importance of classroom indoor air quality (IAQ) and thermal comfort (TC) especially as it pertains to children. Absenteeism rates (or attendance rates), is one of the major issues with which parents and school officials are concerned. Previous studies recognized this issue and suggested more consistent and well-designed research studies to investigate the association between absenteeism and classroom IAQ/TC. This is because “strong study design, including the best available measurements of exposure and human outcomes, are both feasible and necessary for good IEQ research” [1,2].

Previous researchers sought to associate the school absenteeism with IAQ and TC [3,4,5]. Few included multiple environmental factors of classroom IAQ and TC, and even fewer considered the contribution from seasonal variations.

Heating and non-heating seasons were based on the operation status of Heating, Ventilation, and Air-conditioning (HVAC) systems, and the building indoor conditions were majorly influenced by the principle differences of the system operating in heating season comparing to the system of non-heating seasons, according to the ASHRAE Handbook of Fundamentals [6]. First, the characteristics of the primary air from the diffusers were different, such as volumetric flow rates (due to different heating and non-heating load calculation), supply air velocity, set-points for supply air temperature, etc. Second, air diffusion patterns from diffusers were different, for rooms with a ceiling supply, the supply air dropped quickly to the floor during non-heating and remains at (or rises to) ceiling level for a longer period during the heating season. Third, the directions of natural convection between the mixing zones and stagnant zones (if any) were different as hot air rises and cool air sinks. Finally, the general room air flow patterns are also influenced by the temperature of the exterior surface and infiltration (or exfiltration).

Thus, to examine the different influence between IAQ/TC of classroom with illness-related absenteeism (IRA) between heating and non-heating seasons, the research team performed this study in the Midwestern United States. The research team hypothesized that classroom IAQ and TC might influence the students’ IRA, and the seasonal variation (between heating or non-heating) of such influences should clearly be observed. Since the “seasons” defined in this study was from the different operation modes of HVAC system, a naturally ventilated classroom without HVAC system was not discussed.

## 2. Materials and Methods

Schools districts of the Midwest United States, were included in the study. Various IAQ and TC metrics of classrooms were collected from the participating schools. Measurement campaigns, were carried out three times per year, two consecutive days per classroom visit, for each classroom for classroom IAQ and TC factors. The daily IRA and demographic data of students were also collected. A Negative Binomial (NB) statistical model was structured to estimate the association between independent (classroom IAQ and TC factors) and dependent (students’ IRA) variables.

### 2.1. Classroom Selection and Student Data

The research team reached out the school districts from two Midwest metropolitan areas to participant this study during the 2015–2016 school year. The biggest school district of each area was invited, if declined, the second biggest district was invited. In each school district, data was collected from 3rd and 5th grade classrooms of elementary school. Up to three classrooms of each grade. All engaged schools were located in Climate Zone 5A, which was defined as “Cold climate zone” by the Department of Energy [7].

The school districts provided daily absence counts associated with the students in the selected classrooms and the clarification (reason) for the absence. The absence related to the illness (IRA, based on the reason, despite the kinds of illness) were used in this study. The IRA indicated the total count of absence during a certain period. Demographic information of students was also collected to better describe the population of the students in the participating schools.

### 2.2. Environmental Data

In each of the classrooms, a list of IAQ and TC data was collected. The list included measurements of the concentration of indoor carbon dioxide (CO_2_), formaldehyde (HCHO), the count of particulate matter (PN) with aerosol diameters ranging from 0.3 μm to 2.5 μm, air temperature (T), relative humidity (RH), and globe temperature (GT). Various measurement meters were used to collect different environmental data. Custom-built hanging kits and a special housing were used for each classroom to ensure the sensors safely hung from the ceiling and to provide a secure place for sensors at breathing height. The housings were tested for barriers to air movement and to ensure they were out of reach of the students. Four absorption infrared CO_2_ m (the Telaire 7001 by GE Sensing), which had a display resolution of 1 ppm with accuracy of 50 ppm or 5% of the reading (whichever is greater) and detecting range of 0–5000 ppm, were deployed to each classroom. The four CO_2_ m were attached to different locations to represent the supply, return, room averaged, and outdoor concentration of carbon dioxide. The formaldehyde monitor was placed in the special housing (the FM 801 by ShinYei/GrayWolf) and utilized photometric technology and continuously reported the formaldehyde (HCHO) concentration every 30 min. The formaldehyde monitor had a resolution of 1 ppb, tested a 10% accuracy at 40, 80, and 160 ppb, with a range from 10 ppb to 1000 ppb. The optical particle counter (the Handheld 3016 by Lighthouse Worldwide Solutions) had six measuring channels, ranging from 0.3 micrometers (μm) to 10 micrometers. The six channels are 0.3, 0.5, 1.0, 2.5, 5.0, and 10.0 μm. The particulate matters were grouped into PN2.5 (with particles had a diameter less than 2.5 μm). T and RH were collected by the sensors installed inside of the HOBO data logger (the HOBO U12-012 by Onset). The temperature sensor had a resolution of 0.03 °C, an accuracy of 0.35 °C from 0 °C to 50 °C. The humidity sensor had a resolution of 0.05% RH, with an accuracy typically 2.5% from 10% to 90% RH, to a maximum of 3.5% RH including hysteresis at 25 °C. In total four HOBO data logger were implied to each of the classroom and placed as the same manner as the four CO_2_ m. The GT used a thermocouple (the TMC6-HD by Onset), which had an accuracy of 0.25 °C within 0 °C to 50 °C, and a resolution of 0.03 °C at 20 °C and placed inside of a black coated globe (with diameter 40 mm) and connected to the HOBO data logger, was placed to the special housing for each classroom.

**Figure 1:**
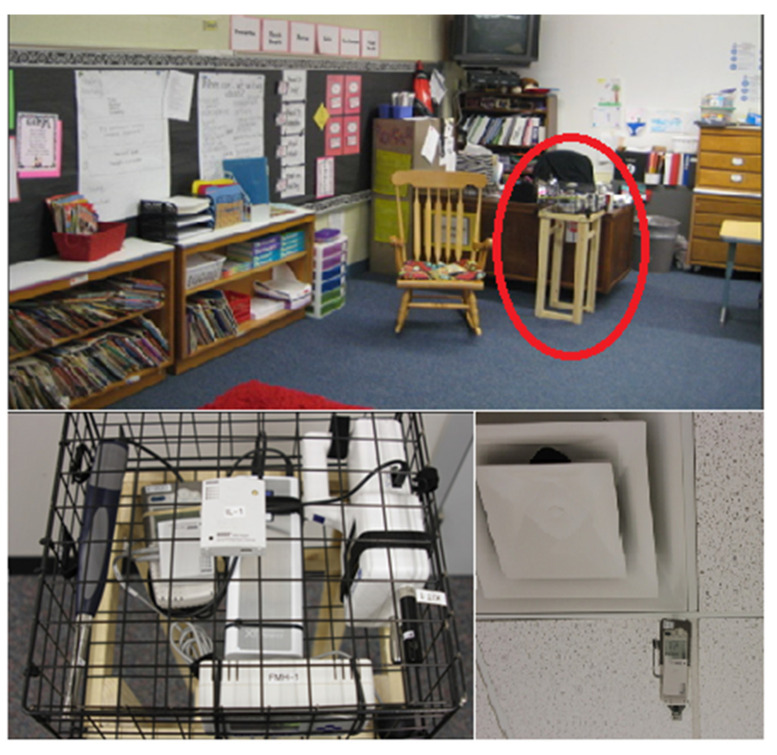
Equipment housing placed next to teacher’s desk (**photo on the top**), close looking of the special housing (**photo at the lower left**), hanging CO_2_ m (**photo at the lower right**) [8].

The data measurement logged data from each classroom for approximately two days, starting from approximately 7:00 a.m. On day one, and ending at approximately 5:30 p.m. on day two. The measurement was repeated three times during the academic year. For the details of the measurement of environmental data, please referring to the Figure 1 and the previous published article by the research team [8].

### 2.3. Heating and Non-Heating Seasons

The heating and non-heating seasons were defined based on the operation status of the HVAC system in each classroom. When the HVAC system was providing heating to the classroom, the days were defined as the heating season. Otherwise, the days were defined as the non-heating season. The heating degree days (HDDs) were calculated based on local weather data and the fact that the school districts sought to maintain an average indoor temperature of 21 °C. The calculated HDDs were used to determine the operation model of HVAC system. Then the heating season was refined based on the measured temperature data. In total, three data loggers were deployed to measure the air temperature at different locations of each classroom. Two of the loggers were attached to the supply air diffuser and return air grill, accordingly. Thus, the temperature difference between the supply and return air was used to confirm the operation status of the HVAC system.

### 2.4. Data Collection and Analysis

The research team collected the continuous data from the two-day period in the occupied measurement plan. However, the research team had chosen the data during the occupied hours only as to develop the analytical model. The occupied hours were defined as the time between the start time of the first class and the end time of the last class of each school day. Thus, the averaged values presented in this paper should better represent the actual exposure that the students and teachers were experienced during their school days.

The research team estimated the ventilation rate (VR) based on the indoor CO_2_ concentration, converted the PN to the mass concentration of particulate matter (PM), converted the RH to absolute humidity (AH). 

The equilibrium CO_2_ analysis approach, which adopted from the ASTM D 6245—12 Standard was used in this study to estimate the indoor ventilation rate. The equilibrium indoor CO_2_ concentration was defined as the 95th percentile of the 15-min moving averaged CO_2_ concentration during the occupied hours. The estimation used the 0.0043 L/s per 3rd and 5th grade students and 0.0051 L/s per adult, and used the actual number of students in each classroom which provided by the school districts plus one adult (teacher) per classroom. 

In addition, in this study, the optical counts of particulate matter, PN2.5, were converted into mass concentration, PM2.5 (mass concentration of particles that had a diameter less than 2.5 μm) by using sphere mass calculation equation, and by using the centroid of the distribution of each of the six size channels as the mass mean diameters [9]. The conversion used the 1.65 g/cm^3^ as the assumed averaged density of PM2.5, which was adopted from reference [10]. 

The research team used SPSS (IBM SPSS Statistics 22, Armonk, NY, USA) to perform the data analysis. First, the descriptive statistics were generated for all IAQ and TC metrics from participating schools. The descriptive statistics and paired sample *T* test were reported on the primary outcome variable (Illness-related absenteeism—IRA) during both heating and non-heating seasons. The daily IRA are count data and highly skewed [11]. The normality test was performed on the daily IRA and confirmed the non-normality of the distribution. Thus, a negative binomial (NB) model was selected. Poisson regression was also considered during the analysis process, as it is a usual way to analyze this type of count data. However, over-dispersion of the data distribution suggested the final selection of the NB model. 

The results of the negative binomial model used the incidence rate ratios (IRR) to estimate the effects. IRR can suggest that for a one-unit increase of the IAQ or TC variables, the IRA is expected to increase (or decrease if the IRR is less than 1) by a factor of IRR. Ordinary least squares regression and Poisson regression were used to validate the results of the NB model.

## 3. Results

In total, 85 classrooms, from 21 elementary of two Midwestern United States school districts (District A and B) were participated in this study. Eleven elementary schools in District A and ten elementary schools in District B were engaged. All engaged classrooms were mechanically ventilated with no operable exterior windows or exterior doors. The descriptive statistics are contained in Table 1. 

### 3.1. Environmental Data

Table 2 presents the descriptive information of the IAQ and TC that were directly collected from each of the classrooms. All the variables are averaged through the occupied hours for each visit and grouped by different seasons (heating or non-heating). Mean, 25th, 50th, 75th, and 95th percentiles were included to indicate the distribution of each variable. Extreme cases, which are defined as outliers and detected based on the interquartile range rule, are excluded from the model.

### 3.2. Model Results

The results of the NB model are shown in Table 3. The IRR was reported for the association of IAQ and TC factors with IRA. The IRR is described as the relative difference between the incidence rates of events. An IRR value of less than 1.000 indicated the decreased IRA was associated with a multiple-unit increase of IAQ and TC factors, vice versa. 

The count of indoor particles, PN2.5 to be more exact, was associated with IRA only during the non-heating season. The predicted increase of the IRA is 3% for each additional 1,000,000-counts/L increase of PN2.5. The association between PM2.5 and IRA had similar trend, but only marginally significant on statistical analysis. 

Indoor CO_2_ concentration was significantly associated with IRA during the heating season only, with a 100 ppm increase in the CO_2_ concentration, the expected increase of IRA is 3%.

## 4. Discussion

The IAQ and TC conditions of 85 classrooms were collected from schools of the Midwestern United States. The interested variables, which averaged during the occupied hours, were compared to the IRA of students from the third and fifth grades.

### 4.1. Primary Findings

In this study, the results indicated that to potentially reduce the IRA in the elementary classrooms by controlling the classroom IAQ and TC, the targeting environmental factors in the heating season were different than those in the non-heating season. On the other word, the operating mode of HVAC systems might significantly influence the IRA of elementary students. 

#### 4.1.1. Ventilation Rate and Indoor CO_2_ Concentration

The ventilation rate (estimated based on the indoor CO_2_ concentration) was significantly associated with student absenteeism in several previous studies. Shendell [4] reported annually averaged absenteeism of 434 U.S. classrooms in 22 schools in the states of Washington and Idaho in the U.S. The relative decrease in absenteeism was 2.1–7.6% when associated with a 1 L/s·person increase of VR. Another comprehensive study which continuously measured the VR of the classroom for a two-year period also reported a 1–1.5% relative decrease of IRA with each additional 1 L/s·person VR [12]. A UK study of 60 naturally ventilated classrooms reported a 100 ppm increase of indoor CO_2_ concentrations, when compared to outdoors, and was associated with 0.2% decrease of annual attendance [5]. A 100-ppm difference in CO_2_ concentration can be translated to VR, which is about 0.48 L/s·person.

Compared to the findings this study, only the heating season showed a significant association which a 3% increase in absenteeism was expected with every additional 100 ppm of CO_2_ concentration. In the meantime, the VR did not show a similar association with IRA as the CO_2_ concentration during the heating season. This new evidence may suggest that, even the indoor ventilation rate was usually estimated based on the indoor CO_2_ concentration, these two factors were not necessary tell the same story. The estimated ventilation rate in this study was based on the equilibrium CO_2_ concentration, which represented the near-peak steady-state condition of CO_2_. Thus, the operational statuses of the HVAC systems and the seasons (i.e., whether it is heating or non-heating) might significantly affect the associations and conclusions from the previous studies based on the annual data (or the data from one season). 

Noted that a greater VR is associated with increased academic performance, regardless of the seasons [5,13,14]. Thus, the future studies that intent to understand the relation between classroom ventilation rate and academic performance, where considering the student absenteeism as mediator in between, might include the seasonal variation in their equation. 

#### 4.1.2. Indoor Particulate Matters

Many prior studies significantly associated the absenteeism of schools with particulate matters in ambient air [15,16,17]. However, few studies had examined the association between absenteeism of schools and indoor particles. A longitudinal study associated the indoor particles with absenteeism in two daycare centers founded the reduced concentration of small particles was significantly associated with decreased absenteeism [18]. 

In current study, PN2.5 was significantly associated with illness-related absenteeism during the non-heating season only, while the PM2.5, which calculated based on the PN2.5, shown only marginally significant association for the same season. The results firstly indicated that the indoor particles might adversely influence the student health (which represented by the illness-related absenteeism of students). However, the difference of associations to health between PN2.5 and PM2.5 potentially suggested that even a small level of mass concentration of indoor particles could significantly influence student health. This potentially suggested that controlling the pathogens infection process, which using the indoor particles as carrier, traveling from one student to another, and resulting a healthy student got disease and absence from school, is still a significant benefit of removing the indoor particles even the indoor particulate matters’ mass concentration is relatively low. 

The VR is neither correlated with PN2.5 nor significantly associated with IRA, indicating that the association between PN2.5 and IRA might not be mediated by the association between VR and IRA. 

### 4.2. Potential Findings-Air Temperature, Relative Humidity, Absolute Humidity, and Indoor Formaldehyde

Several marginally association were observed in this study between IAQ/TC variables with IRA. Significant associations might be observed in future studies if the sample size were increased or studies performed in other locations. Thus, the following discussion might be considered as the supplementary to previous significant finds.

The relative humidity (RH) is an environmental factor which considered as combination of the actual moisture content (or AH) and the air temperature (T). Both AH and T were marginally associated with IRA during non-heating season, however, the RH in the current study has a less significant association with IRA. In an early Canadian study, a statistically significant correlation was observed between school absenteeism and relative humidity [19]. Other than this, there were other studies that either without a significant association between the humidity and absenteeism [20] or, with the small sample size, failed to reach a more general finding [21,22]. The Canadian study recruited 12 schools located in a colder and drier climate zone with a longer winter comparing to the schools in this current study. This difference might indicate that the importance of classroom air temperature and humidity increase for locations with server cold and dry weather. 

In this study, formaldehyde was innovated examined in elementary classroom and associated with illness-related absenteeism. Unfortunately, only marginal association was observed between indoor formaldehyde and IRA during non-heating season. Air temperature and moisture content were identified as influencing factors for the emission rate of formaldehyde [23]; thus, the marginal association might be explained by the correlation among formaldehyde concentration, air temperature, relative humidity, and absolute humidity. Thus, future studies either with expanded sample size or with causal path analysis between formaldehyde and student health were required for formaldehyde-health research.

### 4.3. Strengths, Limitations, and Other Thinkings

The strengths of this study are a wide variety of IAQ and TC factors from these elementary classrooms, student absenteeism data grouped based on “sickness vs. non-sickness”, a relatively large number of classrooms in the Midwestern United States, repeated (three-times) measurements of the IAQ and TC factors, and the seasonal data of heating and non-heating. The findings could potentially apply to many other similar classrooms in the same climate region. Unlike many previous studies, their discussion and conclusion were depended on the subjective data from the occupants’ surveys based on the perceived IAQ and TC conditions. Our study collected many objective data with the scientific grade and “state of the art” instruments. About the detailed discussion of the environmental data itself (IAQ and TC factors), please refer to the previous published article by the research team. 

However, the research team also recognized that there are some limitations in the study. Firstly, the current study only included classrooms at the elementary level. We understood that these elementary classrooms followed a more predictable schedule and a constant class-enrollment when compared to those in secondary education classrooms. Thus, the current conclusions about the adverse association of IAQ and TC conditions and student’s absenteeism may only apply to the elementary classrooms. As in any observational study, we are not allowed to make any causality statement. In addition, the findings presented in this paper were only applicable to the classrooms of similar demographics and similar climate regions with comparable ranges of IAQ and TC conditions.

Another limitation was that, due to the privacy nature of the student’s data, the absenteeism data provided by the school districts were very restricted. The school districts only provided the research team with the daily absence count and primarily two types of absenteeism (sickness or non-sickness), The research team has no ability to further access the students’ absenteeism data, such as the diagnosis of the illness or the structure of the student’s family. 

Although the key objective of this paper is to address the difference between heating and non-heating seasons associated the IAQ and TC factors and their associations with the IRA of the students, the demographic data were also collected to describe the student populations at the schools. In this paper, the research team did not include this in the model yet since we do not expect that the demographic data within the same school would vary much among heating and non-heating seasons within the same academic year. 

The intention of the study is to include as many IAQ and TC variables as possible and to repeat the measurements during heating and non-heating seasons as much as possible. However, due to the limitations of budget and time resources, the occupied measurements were implemented three times in the academic year, with continuous measurement over each two-day visit for each classroom. With 255 classroom visits over the duration of the study, the research team attempted to capture the anticipated seasonal variation for the closest approximation possible. A longer-duration (such as week-long) and continuous sampling (even daily—365 days) were desirable for this type of classroom environmental monitoring in the future. 

## 5. Conclusions

Based on this study, during non-heating season, a 3% increase of illness-related absenteeism was estimated with 1,000,000-counts/L increase of particles that had a diameter less than 2.5 μm (PN2.5); during the heating season, a 3% increase of illness-related absenteeism were expected with 100 ppm increase of room averaged CO_2_ concentration; and these results suggested that the indoor air quality and thermal condition factors could associated with absenteeism related to illness differently between heating and non-heating seasons.

In the studied classrooms, majority (around 80%) were under-ventilated (by 6.5 L/s·person as compared to the suggested value from the ASHRAE Standard 62.1-2010), absenteeism were associated to the elevated CO_2_ concentration in the heating session but not in the non-heating season. Therefore, it is suggested to carried out an experimental study to validate the causal relationships between the classroom indoor air quality/thermal conditions the absenteeism of the students in a more controlled setting, the impacts from CO_2_ concentrations and concentrations of indoor fine particle should firstly be considered. In addition, since the associations of indoor air quality and thermal comfort factors with illness-related absenteeism in elementary classrooms differs with heating and non-heating seasons, the seasonal (multiple) visits are suggested in all future studies. 

## Figures and Tables

**Table 1:** School, classroom, student, and demographic information.

	District A	District B	Full Model Districts
School information			
Number of schools	11	10	21
Number of classrooms	45	40	85
Number of 3rd grade classrooms	25	18	43
Number of 5th grade classrooms	20	22	42
Student information			
Average enrollment per classroom	23	20	22
Enrollment 3rd grade	23	20	22
Enrollment 5th grade	23	20	22
Total enrollment	1033	801	1834
Enrollment of 3rd grade	579	360	939
Enrollment of 5th grade	454	441	895
Average percentage of free/reduced lunch	58.66%	32.71%	52.95%
Average percentage of White	75.31%	73.7%	74.6%
Average percentage of English Leaner	10.94%	1.88%	6.98%
Average percentage of special education	14.23%	14.46%	14.33%
Average percentage of gifted/talented	13.75%	2.27%	8.73%
Absenteeism			
Total IRA days of the academic year 2015–2016	3844	5154	8998
Averaged daily IRA of the academic year	21.7	28.8	25.3
Averaged daily IRA of heating season	18.6	23.4	21.0
Averaged daily IRA of non-heating season	24.2	33.2	28.7

**Table 2:** Indoor air quality (IAQ) and thermal comfort (TC) data.

	Heating Season					Non-Heating Season				
Percentile (%)	25th	50th	75th	95th	Mean	25th	50th	75th	95th	Mean
PN2.5 (count/L)	15,526	24,376	32,545	53,222	29,147	17,680	22,478	31,976	64,687	31,651
PM2.5(µg/m^3^)	2.56	3.28	4.28	7.44	3.95	2.50	3.24	4.06	8.55	4.36
CO_2_ (ppm)	916	1142	1371	1599	1152	814	1040	1246	1468	1035
HCHO (ppb)	5	5	5	5.19	5.08	5	6.2	9.1	16.41	7.8
VR (L/s·person)	3.60	4.55	6.30	10.42	5.23	3.72	4.94	7.10	11.21	5.70
T (°C)	21.9	22.4	23.3	24.2	22.4	21.7	22.4	23.1	23.8	22.3
RH (%)	25.1	29.2	34.1	37.7	29.7	41.5	48.5	57.0	67.5	50.0
AH (g/m^3^)	5.52	5.84	6.86	7.46	5.95	8.3	9.1	11.2	13.3	9.7
GT (°C)	19.1	20.1	21.6	27.7	20.9	19.1	19.7	22.4	37.38	21.49

**Table 3:** Estimated negative binomial model of log transformed illness-related absence.

	Heating Season		Non-Heating Season	
Variables	IRR	*p*-Value	IRR	*p*-Value
PM2.5 (µg/m^3^)	1.014	0.181	1.012	0.085
PN2.5 (count/L)	1.000	0.203	1.000 *	0.009 *
VR (L/s·person)	0.982	0.259	1.006	0.732
CO_2_ (ppm)	1.0003 *	0.042 *	1.000	0.433
HCHO (ppb)	0.915	0.394	1.022	0.069
T (°C)	1.049	0.159	1.094	0.058
RH (%)	1.003	0.720	1.005	0.184
AH (g/m^3^)	1.028	0.479	1.038	0.064
GT (°C)	0.998	0.894	1.003	0.917

* Significantly associated with a log-transformed count of IRA with significance level of 0.05.
